# Methyl 3-[(1,1-dioxo-1λ^6^,2-benzothiazol-3-yl)amino]-5-nitrothiophene-2-carboxyl­ate

**DOI:** 10.1107/S1600536812038378

**Published:** 2012-09-19

**Authors:** Haridas B. Rode, Jarosław Chojnacki, Hans-Hartwig Otto

**Affiliations:** a Department of Pharmaceutical/Medicinal Chemistry, Institute of Pharmacy, Ernst-Moritz-Arndt-University, Friedrich-Ludwig-Jahn-Str. 17, Greifswald, D-17489, Germany; b Council Scientific and Industrial Research (CSIR) Head Quarter, Anusandhan Bhavan, 2 Rafi Marg, Delhi-110001, India; c Chemical Faculty, Gdańsk University of Technology, G. Narutowicza 11/12, PL-80233 Gdańsk, Poland

## Abstract

The title nitro­thio­phene compound, C_13_H_9_N_3_O_6_S_2_, crystallizes with two independent mol­ecules in the asymmetric unit; the mol­ecular structure of each is stabilized by an intra­molecular N—H⋯O hydrogen bond. The two mol­ecules adopt flattened but slightly different conformations, *viz*. the dihedral angle between the thio­phene ring and the essentailly planar 1,2-benzisothia­zole fragment (r.m.s. deviations = 0.0227 and 0.0108 Å, respectively) is 15.62 (11)° in one mol­ecule and 5.46 (11)° in the other. In the crystal, mol­ecules are arranged into layers parallel to (-111) with weak C_ar_—H⋯O inter­actions formed within the layer. N—H⋯O hydrogen bonds also occur. There are π–π stacking inter­actions between the mol­ecules in neighbouring layers, the distance between the centroids of the 1,2-benzisothia­zole benzene rings being 3.8660 (16) Å. Moreover, dipolar S=O⋯C=O inter­actions with an O⋯C distance of 2.893 (3) Å are observed between the symmetry-independent mol­ecules in different layers. The title compound showed weak inhibition of HLE (human leukocyte elastase).

## Related literature

For general information on elastases, see: Bode *et al.* (1989 ▶); Edwards & Bernstein (1994 ▶). For biochemical assays of HLE inhibition, see: Rode *et al.* (2005 ▶, 2006 ▶). For information on the synthesis, see: Wade *et al.* (1979 ▶); Gupta *et al.* (1999 ▶).
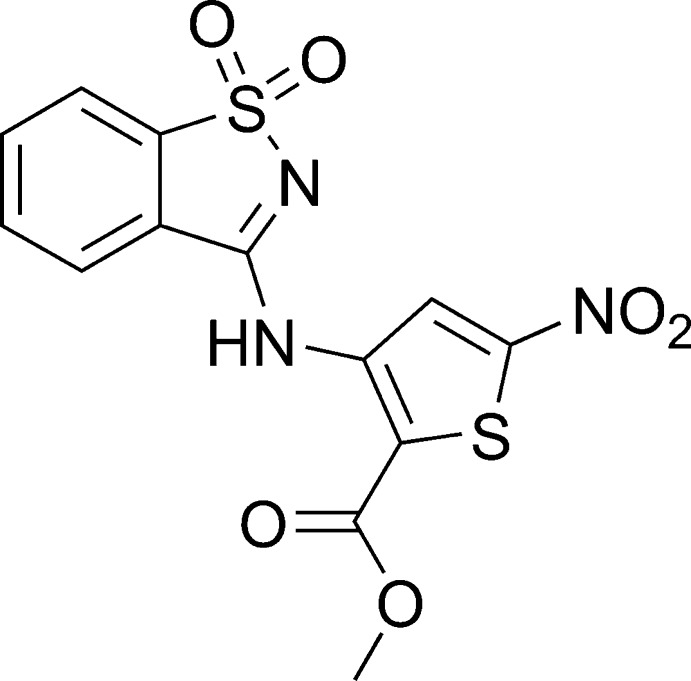


## Experimental

### 

#### Crystal data

C_13_H_9_N_3_O_6_S_2_
*M* *_r_* = 367.35
Triclinic, 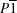

*a* = 8.4462 (7) Å
*b* = 12.5495 (11) Å
*c* = 15.2557 (14) Å
α = 100.754 (7)°
β = 96.956 (7)°
γ = 105.287 (7)°
*V* = 1507.5 (2) Å^3^
*Z* = 4
Mo *K*α radiation
μ = 0.39 mm^−1^
*T* = 293 K
0.44 × 0.29 × 0.12 mm

#### Data collection

Kuma Diffraction KM4CCD Sapphire2 diffractometer
14750 measured reflections
8613 independent reflections
7242 reflections with *I* > 2σ(*I*)
*R*_int_ = 0.033

#### Refinement

*R*[*F* ^2^ > 2σ(*F* ^2^)] = 0.060
*wR*(*F* ^2^) = 0.165
*S* = 1.09
8613 reflections
443 parameters
H atoms treated by a mixture of independent and constrained refinement
Δρ_max_ = 0.71 e Å^−3^
Δρ_min_ = −0.32 e Å^−3^

### 

Data collection: *CrysAlis PRO* (Oxford Diffraction, 2009 ▶); cell refinement: *CrysAlis PRO*; data reduction: *CrysAlis PRO*; program(s) used to solve structure: *SUPERFLIP* (Palatinus & Chapuis, 2007 ▶); program(s) used to refine structure: *SHELXL97* (Sheldrick, 2008 ▶); molecular graphics: *ORTEP-3* (Farrugia, 1997 ▶); software used to prepare material for publication: *publCIF* (Westrip, 2010 ▶), *PLATON* (Spek, 2003 ▶), *WinGX* (Farrugia, 1999 ▶) and *Mercury* (Macrae *et al.*, 2006 ▶).

## Supplementary Material

Crystal structure: contains datablock(s) global, I. DOI: 10.1107/S1600536812038378/gk2503sup1.cif

Structure factors: contains datablock(s) I. DOI: 10.1107/S1600536812038378/gk2503Isup2.hkl

Supplementary material file. DOI: 10.1107/S1600536812038378/gk2503Isup3.cml

Additional supplementary materials:  crystallographic information; 3D view; checkCIF report

## Figures and Tables

**Table 1:** Hydrogen-bond geometry (Å, °)

*D*—H⋯*A*	*D*—H	H⋯*A*	*D*⋯*A*	*D*—H⋯*A*
N2—H2*A*⋯O5	0.88 (3)	2.08 (3)	2.765 (2)	134 (3)
N5—H5*A*⋯O11	0.84 (3)	2.17 (3)	2.825 (3)	134 (3)
N5—H5*A*⋯O2^i^	0.84 (3)	2.55 (3)	3.141 (3)	128 (3)
C4—H4⋯O4^ii^	0.93	2.48	3.376 (3)	162
C5—H5⋯O10^ii^	0.93	2.51	3.316 (3)	146
C17—H17⋯O3^iii^	0.93	2.38	3.223 (3)	151

Symmetry codes: (i) 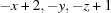; (ii) 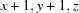; (iii) 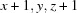.

**Fig. 1.**
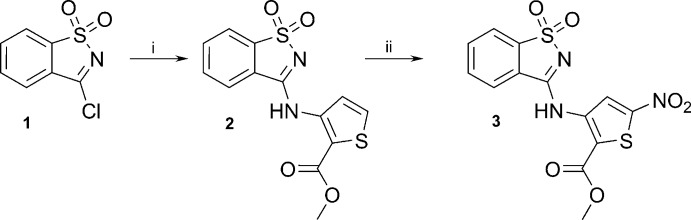
Reagents and conditions: (i) methyl 3-aminothiophene-2-carboxylate, dioxane, reflux, 2 h; (ii) conc. H2SO4, conc. HNO3, -30 °C, 45 min.

**Fig. 2.**
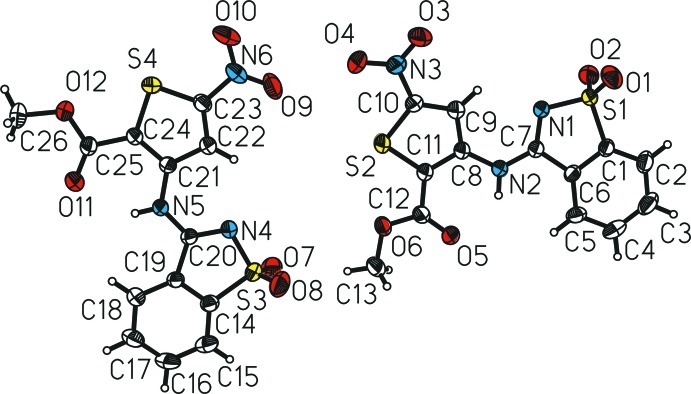
Molecular structure and labeling scheme for 3. Displacement ellipsoids are drawn at the 50% probability level.

**Fig. 3.**
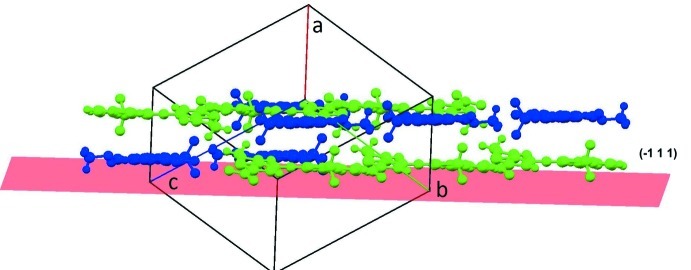
Crystal packing of the title compound. The two symmetry independent molecules (coloured green and blue) bound by Car-H···O interactions form layers parallel to (-1 1 1).

## supplementary crystallographic information

### Comment

Elastases are endopeptidases (serine proteases) which by definition are able to solubilize elastin by proteolytic cleavage and are possibly the most destructive enzymes in the human body, having the ability to degrade virtually all connective tissue components (Bode *et al.* 1989). Uncontrolled proteolytic degradation by elastase has been implicated in a number of pathological conditions including ARDS (Adult Respiratory Distress Syndrome) and lung injury, cystic fibrosis, pulmonary emphysema, smoking related chronic bronchitis and rheumatoid arthritis (Edwards *et al.* 1994). Therefore considerable research has been focused on developing potent inhibitors or drugs against HLE (Human Leukocyte Elastase). Potent elastase inhibitors are based on peptidic, heterocyclic and non-heterocyclic scaffolds. Of interest are the heterocyclic inhibitors as these small molecules potentially offer advantages over the larger, peptide based inhibitors due to their increased proteinase stability, increased oral absorption and decreased structural complexity. In our earlier reports, we described peptidic and heterocyclic elastase inhibitors and illustrated that the pseudosaccharin amine derivatives are potential inhibitors of elastase (Rode *et al.* 2005, Rode *et al.* 2006). Pseudosaccharin amines were further explored to synthesize analogues containing thiophene and thiazole components. During the nitration of the thiophene analogue 2 (see Figure 1), we observed the electrophilic attack of a nitro group at the α - position of thiophene to produce 3. Here we report the structural assignment of the thiophene derivative 3 using NMR spectroscopy and single crystal XRD. Compound 3 show weak inhibition of PPE (Porcine Pancreatic Elastase) and HLE.

The synthesis of pseudosaccharin chloride 1 (see Figure 1) was carried out according to the literature procedure (Wade *et al.*, 1979). The reaction between 1 and methyl 3-aminothiophene-2-carboxylate resulted in a brown colored solid 2. This solid was treated in a nitrating mixture at -30 °C yielding C_13_H_9_N_3_O_6_S_2_, 3. The compound was further analysed as decribed below.

Compound 3 crystallizes with two independent molecules in the asymmetric unit (*Z*=4). An *ORTEP* view of the asymmetric unit is shown in Figure 2. The molecules are chemically identical and the most significant differences are in dihedral angles between related NO_2_ groups and the aromatic ring and dihedral angle between thiophene and benzene mean planes. To be more specific: the torsion angles differ by ca. 14° [C9—C10—N3—O3 -9.3 (4) ° and C22—C23—N6—O9 4.9 (4)°] and the dihedral angles by ca. 10° [benzene C1-C6 and thiophene C8-C11-S2 form angle 15.50 (13)°, the related rings 5.41 (13)°]. Apart from that the molecules are very similar, overlay of the molecules by fitting all 24 non-hydrogen atoms gives mean r.m.s. deviation of 0.217 Å with maximum distance of 0.552 Å between O9 and O3 in nitro groups (Mercury 3.0, Macrae *et al.* 2006).

The crystal packing is presented in Figure 3. Both molecules are placed in a layer parallel to the (-1 1 1) plane. Such planes spread throughout the crystal forming specific packing pattern. The only intramolecular hydrogen bonds are N2—H···O5 and N5—H···O11 in both molecules, respectively (Table 1). There are also weak C(aromatic)—H···O interactions between the molecules ( Table 1). One can also expect stacking interactions between the aromatic rings, but analysis with *PLATON* (Spek, 2003) reveals that most of the rings are too far away. The closest benzene rings C14–C19 are related by the symmetry center at 3/2, 0, 1 and their centroids are separated by the distance of 3.8660 (16) Å with perpendicular distance between the planes of 3.5883 (11) Å. Other ring centroids are separated by more than 4 Å. Noteworthy, short O1···C25^i^ and O9^ii^···C12 contacts resemble transient states in early stages of the nucleophilic attack of negatively charged oxygen atoms on the partly positively loaded carbon atom in carbonyl groups.

General description and spectral properties

In the ^1^H NMR spectrum of 3, a sharp singlet appeared at δ 8.55 p.p.m. assignable to a proton of the thiophene ring. Information obtained from ^1^H NMR, ^13^C NMR, DEPT, HMQC and HMBC reveals that a carbon at δ 125.58 p.p.m. is assignable to a thiophene carbon bearing a proton, while carbons at δ 123.06, 133.65, 134.30, 121.74 p.p.m. are assignable to CH of the pseudosaccharin scaffold. A coupling is observed between a proton of the pseudosaccharin scaffold and a carbon at δ 157 p.p.m., allowing us to assign δ 157 p.p.m. for the carbon of C=N. Also a coupling between the methoxy group signal at δ 3.93 p.p.m. of thiophene scaffold and that of the carbonyl group at δ 161 p.p.m. was observed in HMBC. No coupling was observed between a proton at δ 8.55 p.p.m. (thiophene bearing proton) and any of the carbons of thiophene ring. If both the nitro-isomers *i.e.* 4-nitro analog (structure not shown) and 5-nitro analog (3) could have been isolated, that would have helped to solve the structure of 3 based on the relative chemical shifts. But only one isomer was obtained. Although the possibility of formation of the other isomer cannot be ruled out as in the ^1^H NMR spectrum of the crude product, more products were indicated but these products could easily be neglected in crystallization and only one isomer was obtained as a major product. Therefore the structure of the nitro thiophene analogue was determined by X-ray crystallography and in fact the product was found to be the 5'-nitro analogue 3. It is important to note that the thiophene undergoes electrophilic substitution reactions slowly and selectively at an α-position to sulfur rather than at β-position. The preferential electrophilic attack at an α-position in thiophene may be explained on the basis of stability of the transition state (Gupta *et al.*, 1999).

Compound 3 was tested for its ability to inhibit PPE (Porcine Pancreatic Elastase) and HLE (Human Leukocyte Elastase) activity in the biochemical assay. More information on elastase can be found elsewhere (e.g. Bode *et al.* (1989); Edwards & Bernstein, 1994; Rode *et al.*, 2005). The detailed description of these biochemical assays is reported in our earlier work (Rode *et al.*, 2006). It is important to note that compound 3 inhibited 32% activity of PPE at 100 µ*M* concentration and 15% activity of HLE at 200 µ*M* concentrations. Although 3 has shown weak inhibition of HLE and PPE, it may serve as a starting point for developing potent HLE inhibitors.

### Experimental

Synthesis of 2

3-Chlorobenzo[*d*]isothiazole 1,1-dioxide 1 (Figure 1) (3.00 g, 14.88 mmol) and 2.33 g of methyl 3-aminothiophene-2-carboxylate (14.88 mmol) were refluxed in 70 ml of dioxane. After cooling to room temperature, the solid was filtered off and washed with a little acetone. The solid was dried to give the analytically pure compound. Yield: 3.96 g (83%); M.p. 284–287°C, Rf = 0.87 (AcOEt/PE 8:2).

Synthesis of 3

To a cooled (-30 °C) and stirred solution of 2 (2.00 g, 6.20 mmol) in 95% H_2_SO_4_ (10 ml), 2 ml of concentrated HNO_3_ were added. The mixture was stirred at -30 °C for 45 minutes and allowed to warm to room temperature. The viscous liquid was poured on ice (10 g) and the resulting aq. phase extracted with dichloromethane (3 × 100 ml). The organic phase was separated, dried with sodium sulfate and evaporated *in vacuo*. The solid was crystallized from dichloromethane: methanol (9:1). Yield: 0.40 g (18%); M.p. 285–287°C (MeOH/CH_2_Cl_2_); *R*_f_ = 0.80 (AcOEt/PE 8:2).

See '_exptl_special_details' in the cif file for more information.

### Refinement

The positions of C-bound H atoms were calculated geometrically and refined in a riding model approximation with C-H bond lengths in the range 0.93–0.96 Å and *U*_iso_(H) = 1.4*U*_eq_(C). The amino hydrogen atoms H2A and H5A were found from difference Fourier maps and freely refined.

### Crystal data

**Table:**

C_13_H_9_N_3_O_6_S_2_	*Z* = 4
*M**_r_* = 367.35	*F*(000) = 752
Triclinic, *P*1	*D*_x_ = 1.619 Mg m^−^^3^
Hall symbol: -P 1	Melting point = 558–560 K
*a* = 8.4462 (7) Å	Mo *K*α radiation, λ = 0.71073 Å
*b* = 12.5495 (11) Å	Cell parameters from 4406 reflections
*c* = 15.2557 (14) Å	θ = 2–30°
α = 100.754 (7)°	µ = 0.39 mm^−^^1^
β = 96.956 (7)°	*T* = 293 K
γ = 105.287 (7)°	Plate, light yellow
*V* = 1507.5 (2) Å^3^	0.44 × 0.29 × 0.12 mm

### Data collection

**Table:**

Kuma Diffraction KM4CCD Sapphire2 diffractometer	7242 reflections with *I* > 2σ(*I*)
Graphite monochromator	*R*_int_ = 0.033
Detector resolution: 8.1883 pixels mm^-1^	θ_max_ = 30°, θ_min_ = 2.6°
ω scans	*h* = −11→11
14750 measured reflections	*k* = −17→17
8613 independent reflections	*l* = −21→20

### Refinement

**Table:**

Refinement on *F*^2^	Primary atom site location: structure-invariant direct methods
Least-squares matrix: full	Secondary atom site location: difference Fourier map
*R*[*F*^2^ > 2σ(*F*^2^)] = 0.060	Hydrogen site location: inferred from neighbouring sites
*wR*(*F*^2^) = 0.165	H atoms treated by a mixture of independent and constrained refinement
*S* = 1.09	*w* = 1/[σ^2^(*F*_o_^2^) + (0.0822*P*)^2^ + 0.8507*P*] where *P* = (*F*_o_^2^ + 2*F*_c_^2^)/3
8613 reflections	(Δ/σ)_max_ = 0.006
443 parameters	Δρ_max_ = 0.71 e Å^−^^3^
0 restraints	Δρ_min_ = −0.32 e Å^−^^3^

### Special details

**Table:**

Experimental. Column chromatography (CC) was performed by using Merck silica gel 60, Nr. 7734. Melting points (M.p.) were determined by MEL-TEMP (Mel-Temp laboratories Inc, USA) melting point apparatus and are uncorrected. Analytical TLC was performed on Merck TLC plates (Aluminium plates coated with silica gel 60 F254, No. 5554). Visualization of spots was carried out by using ultraviolet illumination (λ = 254 nm) and analytical data are reported as "ratio of front"-values (R_f_ -value). Infrared spectra were obtained by using an IR spectrophotometer, Perkin-Elmer 1600 series FTIR. Absorption is reported in relation to wavenumber (¯ν). ^1^H NMR spectra were measured with a Bruker DPX 200 (200 MHz) spectrometer, and ^13^C NMR spectra were measured with a Bruker DPX 200 (50 MHz), both at 25° C with tetramethylsilane (TMS) as an internal standard. Chemical shifts are reported as parts per million (p.p.m., δ units). Coupling constants are reported in Hz. The spectra were analysed by MESTREC NMR software. The following abbreviations were used -s: singlet, bs: broad singlet, d: doublet, m: multiplet. Analytical purity was assessed at 30° C by RP-HPLC using LaChrom apparatus series 7000, Merck Hitachi (Pump: L-7100, Diode-Array-Detector L-7450, Auto sampler L-7200, thermostat column L-7350, Solvent degasser L-7612, Interface D-7000). Column used was LiChrospher 250–4, RP-18, 5 µm. The measurement was carried out at λ max 220 nm unless otherwise stated. All solvents were used without further purification. 3-Aminothiophene-2-carboxylic acid methyl ester was purchased from Aldrich. PPE (EC 3.4.21.36, ≈200 U/mg) and HLE (EC 3.4.21.37, ≈34 U/mg) were purchased from Serva. Suc-(Ala)_3_-pNA, and N-methoxysuccinyl-(Ala)2 –Pro-Val-pNA were obtained from Bachem. compound 2 IR: ¯ν = 3446 (NH), 3090, 2990, 2944 (CH), 1683 (ester with intramolecular hydrogen bonds), 1616 (C=N), 1320, 1161 (SO_2_); ^1^H NMR [D_6_]DMSO: δ = 11.07 (bs, 1H, NH), 8.19–8.10, 8.04–7.90 (2 m, 4H, ar), 8.09 (d, J = 5.40 Hz, 1H, 5'-Hthiophene), 7.85 (d, J = 5.40 Hz, 1H, 4'-Hthiophene), 3.89 (s, 3H, OMe); ^13^C NMR [D_6_]DMSO: δ = 162.84, 156.30, 140.94, 140.83, 134.18, 133.72, 133.08, 127.12, 124.15, 122.63, 121.81, 116.71, 52.46; HPLC: k' = 4.57, t_0_ = 1.85 (RP-18, ACN/ H_2_O 1:1). compound 3 IR: ¯ν = 2958 (CH), 1707 (C=O), 1610 (C=N), 1344, 1174 (SO_2_); ^1^H NMR [D_6_]DMSO: δ = 11.20 (bs, 1H, NH), 8.55 (s, 1H, 4'-Hthiophene), 8.28–8.19, 8.18–8.10, 8.01–7.92 (3 m, 4H, ar), 3.93 (s, 3H, OMe); ^13^C NMR [D_6_]DMSO: δ = 160.98, 157.37, 152.00, 141.04, 137.58, 134.42, 133.79, 126.72, 125.50, 123.50, 123.11, 121.89, 53.37; HPLC: k' = 4.60, t_0_ = 1.77 (RP-18, ACN/ H_2_O 1:1).
Geometry. All e.s.d.'s (except the e.s.d. in the dihedral angle between two l.s. planes) are estimated using the full covariance matrix. The cell e.s.d.'s are taken into account individually in the estimation of e.s.d.'s in distances, angles and torsion angles; correlations between e.s.d.'s in cell parameters are only used when they are defined by crystal symmetry. An approximate (isotropic) treatment of cell e.s.d.'s is used for estimating e.s.d.'s involving l.s. planes.
Refinement. Refinement of *F*^2^ against ALL reflections. The weighted *R*-factor *wR* and goodness of fit *S* are based on *F*^2^, conventional *R*-factors *R* are based on *F*, with *F* set to zero for negative *F*^2^. The threshold expression of *F*^2^ > σ(*F*^2^) is used only for calculating *R*-factors(gt) *etc*. and is not relevant to the choice of reflections for refinement. *R*-factors based on *F*^2^ are statistically about twice as large as those based on *F*, and *R*- factors based on ALL data will be even larger.

### Fractional atomic coordinates and isotropic or equivalent isotropic displacement parameters (Å^2^)

**Table:**

	*x*	*y*	*z*	*U*_iso_*/*U*_eq_	
S1	0.78764 (6)	0.38838 (4)	0.14560 (4)	0.03408 (13)	
S2	0.82812 (8)	0.09330 (5)	0.48592 (4)	0.04228 (15)	
N1	0.7960 (2)	0.32182 (15)	0.22894 (13)	0.0378 (4)	
N2	0.9528 (2)	0.35411 (15)	0.37411 (12)	0.0355 (4)	
N3	0.5301 (3)	−0.00535 (17)	0.37232 (14)	0.0422 (4)	
O1	0.6285 (2)	0.40847 (18)	0.12949 (14)	0.0516 (4)	
O2	0.8372 (2)	0.32854 (15)	0.06896 (12)	0.0482 (4)	
O3	0.4174 (2)	−0.00021 (18)	0.31698 (13)	0.0553 (5)	
O4	0.5245 (3)	−0.08320 (18)	0.41086 (16)	0.0677 (6)	
O5	1.1821 (2)	0.38075 (15)	0.52810 (12)	0.0465 (4)	
O6	1.1375 (2)	0.23414 (16)	0.59779 (12)	0.0475 (4)	
C1	0.9457 (3)	0.51416 (17)	0.20129 (15)	0.0328 (4)	
C2	1.0033 (3)	0.6138 (2)	0.17343 (19)	0.0463 (5)	
H2	0.9595	0.6231	0.1174	0.065*	
C3	1.1305 (4)	0.6994 (2)	0.2336 (2)	0.0574 (7)	
H3	1.1714	0.7683	0.2179	0.080*	
C4	1.1979 (4)	0.6846 (2)	0.3168 (2)	0.0528 (6)	
H4	1.2854	0.7426	0.3546	0.074*	
C5	1.1361 (3)	0.58433 (19)	0.34400 (16)	0.0399 (5)	
H5	1.1799	0.5748	0.4000	0.056*	
C6	1.0071 (2)	0.49860 (16)	0.28539 (14)	0.0314 (4)	
C7	0.9133 (2)	0.38550 (17)	0.29652 (14)	0.0310 (4)	
C8	0.8755 (3)	0.25233 (17)	0.39677 (14)	0.0319 (4)	
C9	0.7199 (3)	0.17426 (18)	0.35328 (15)	0.0361 (4)	
H9	0.6530	0.1811	0.3028	0.050*	
C10	0.6826 (3)	0.08730 (18)	0.39657 (15)	0.0369 (4)	
C11	0.9476 (3)	0.21983 (18)	0.47066 (14)	0.0350 (4)	
C12	1.1014 (3)	0.2871 (2)	0.53436 (15)	0.0375 (4)	
C13	1.2858 (3)	0.2927 (3)	0.66588 (18)	0.0529 (6)	
H13A	1.3808	0.3111	0.6367	0.074*	
H13B	1.3026	0.2446	0.7060	0.074*	
H13C	1.2723	0.3612	0.7000	0.074*	
H2A	1.036 (4)	0.401 (3)	0.416 (2)	0.057 (9)*	
S3	1.19939 (7)	0.08057 (5)	0.84081 (4)	0.03976 (14)	
S4	0.55736 (7)	−0.42292 (5)	0.68575 (4)	0.03977 (14)	
N4	1.0485 (2)	−0.04004 (16)	0.80561 (13)	0.0387 (4)	
N5	0.9233 (2)	−0.19586 (15)	0.86273 (12)	0.0332 (3)	
N6	0.5555 (3)	−0.2926 (2)	0.56298 (13)	0.0463 (5)	
O7	1.3266 (3)	0.08190 (17)	0.78639 (13)	0.0556 (5)	
O8	1.1299 (3)	0.17360 (16)	0.84891 (16)	0.0604 (5)	
O9	0.6148 (3)	−0.2076 (2)	0.53579 (13)	0.0627 (6)	
O10	0.4332 (3)	−0.3710 (2)	0.52161 (15)	0.0746 (7)	
O11	0.7795 (2)	−0.38408 (16)	0.93357 (12)	0.0487 (4)	
O12	0.6092 (2)	−0.53493 (14)	0.82870 (12)	0.0441 (4)	
C14	1.2651 (3)	0.05876 (18)	0.94846 (14)	0.0352 (4)	
C15	1.3942 (3)	0.1271 (2)	1.01748 (18)	0.0474 (6)	
H15	1.4612	0.1963	1.0117	0.066*	
C16	1.4187 (3)	0.0876 (3)	1.09528 (18)	0.0541 (7)	
H16	1.5047	0.1307	1.1428	0.076*	
C17	1.3169 (4)	−0.0153 (2)	1.10338 (17)	0.0516 (6)	
H17	1.3359	−0.0397	1.1565	0.072*	
C18	1.1866 (3)	−0.0831 (2)	1.03386 (15)	0.0394 (5)	
H18	1.1185	−0.1519	1.0398	0.055*	
C19	1.1628 (2)	−0.04395 (16)	0.95559 (13)	0.0302 (4)	
C20	1.0386 (2)	−0.09623 (16)	0.86997 (13)	0.0302 (4)	
C21	0.7995 (2)	−0.25694 (17)	0.78739 (13)	0.0301 (4)	
C22	0.7594 (3)	−0.21886 (18)	0.70763 (14)	0.0337 (4)	
H22	0.8117	−0.1487	0.6967	0.047*	
C23	0.6318 (3)	−0.30199 (19)	0.64987 (14)	0.0355 (4)	
C24	0.7004 (2)	−0.36494 (17)	0.78475 (13)	0.0317 (4)	
C25	0.7026 (3)	−0.42710 (18)	0.85771 (15)	0.0351 (4)	
C26	0.5853 (5)	−0.6019 (3)	0.8964 (2)	0.0609 (8)	
H26A	0.6918	−0.5963	0.9303	0.085*	
H26B	0.5291	−0.6797	0.8671	0.085*	
H26C	0.5191	−0.5739	0.9368	0.085*	
H5A	0.936 (4)	−0.233 (3)	0.902 (2)	0.052 (8)*	

### Atomic displacement parameters (Å^2^)

**Table:**

	*U*^11^	*U*^22^	*U*^33^	*U*^12^	*U*^13^	*U*^23^
S1	0.0325 (2)	0.0321 (2)	0.0353 (2)	0.00579 (18)	−0.00127 (19)	0.01206 (19)
S2	0.0439 (3)	0.0377 (3)	0.0409 (3)	0.0013 (2)	−0.0005 (2)	0.0190 (2)
N1	0.0393 (9)	0.0300 (8)	0.0371 (9)	−0.0008 (7)	−0.0044 (7)	0.0136 (7)
N2	0.0383 (9)	0.0300 (8)	0.0320 (8)	0.0002 (7)	−0.0011 (7)	0.0111 (7)
N3	0.0435 (10)	0.0370 (9)	0.0382 (9)	−0.0006 (8)	0.0040 (8)	0.0093 (8)
O1	0.0346 (8)	0.0658 (12)	0.0582 (11)	0.0152 (8)	0.0018 (8)	0.0270 (9)
O2	0.0598 (11)	0.0430 (9)	0.0405 (9)	0.0169 (8)	0.0036 (8)	0.0071 (7)
O3	0.0434 (10)	0.0597 (11)	0.0490 (10)	−0.0026 (8)	−0.0065 (8)	0.0141 (9)
O4	0.0666 (13)	0.0477 (11)	0.0740 (14)	−0.0122 (9)	−0.0064 (11)	0.0319 (10)
O5	0.0420 (9)	0.0400 (8)	0.0485 (9)	−0.0029 (7)	−0.0049 (7)	0.0181 (7)
O6	0.0429 (9)	0.0492 (9)	0.0435 (9)	−0.0013 (7)	−0.0057 (7)	0.0241 (8)
C1	0.0320 (9)	0.0275 (9)	0.0392 (10)	0.0077 (7)	0.0046 (8)	0.0111 (7)
C2	0.0520 (14)	0.0355 (11)	0.0557 (14)	0.0125 (10)	0.0100 (11)	0.0210 (10)
C3	0.0613 (16)	0.0294 (11)	0.0760 (19)	0.0017 (11)	0.0118 (14)	0.0160 (12)
C4	0.0490 (14)	0.0309 (11)	0.0650 (16)	−0.0022 (10)	0.0071 (12)	0.0001 (11)
C5	0.0362 (10)	0.0322 (10)	0.0421 (11)	0.0025 (8)	0.0002 (9)	0.0008 (8)
C6	0.0313 (9)	0.0246 (8)	0.0370 (10)	0.0069 (7)	0.0047 (7)	0.0066 (7)
C7	0.0313 (9)	0.0276 (9)	0.0336 (9)	0.0067 (7)	0.0039 (7)	0.0098 (7)
C8	0.0326 (9)	0.0311 (9)	0.0312 (9)	0.0056 (7)	0.0045 (7)	0.0111 (7)
C9	0.0360 (10)	0.0347 (10)	0.0349 (10)	0.0059 (8)	0.0023 (8)	0.0108 (8)
C10	0.0375 (10)	0.0322 (10)	0.0350 (10)	0.0012 (8)	0.0023 (8)	0.0091 (8)
C11	0.0352 (10)	0.0326 (9)	0.0346 (10)	0.0036 (8)	0.0026 (8)	0.0127 (8)
C12	0.0363 (10)	0.0397 (11)	0.0328 (10)	0.0042 (8)	0.0019 (8)	0.0121 (8)
C13	0.0490 (14)	0.0580 (15)	0.0436 (13)	0.0027 (11)	−0.0078 (11)	0.0210 (11)
S3	0.0438 (3)	0.0320 (3)	0.0403 (3)	0.0034 (2)	0.0067 (2)	0.0123 (2)
S4	0.0376 (3)	0.0380 (3)	0.0345 (3)	0.0007 (2)	−0.0030 (2)	0.0068 (2)
N4	0.0412 (10)	0.0360 (9)	0.0343 (9)	0.0026 (7)	−0.0003 (7)	0.0137 (7)
N5	0.0322 (8)	0.0319 (8)	0.0314 (8)	0.0028 (6)	−0.0027 (6)	0.0118 (7)
N6	0.0504 (12)	0.0574 (12)	0.0307 (9)	0.0198 (10)	−0.0020 (8)	0.0094 (8)
O7	0.0558 (11)	0.0549 (11)	0.0510 (10)	0.0011 (9)	0.0198 (9)	0.0150 (9)
O8	0.0725 (14)	0.0395 (9)	0.0751 (14)	0.0190 (9)	0.0133 (11)	0.0238 (9)
O9	0.0863 (16)	0.0675 (13)	0.0424 (10)	0.0283 (12)	0.0063 (10)	0.0274 (9)
O10	0.0654 (14)	0.0885 (17)	0.0489 (11)	0.0051 (12)	−0.0229 (10)	0.0113 (11)
O11	0.0500 (10)	0.0497 (10)	0.0387 (8)	0.0017 (8)	−0.0034 (7)	0.0169 (7)
O12	0.0536 (10)	0.0334 (8)	0.0416 (8)	0.0048 (7)	0.0038 (7)	0.0141 (6)
C14	0.0335 (10)	0.0330 (10)	0.0346 (10)	0.0047 (8)	0.0045 (8)	0.0049 (8)
C15	0.0419 (12)	0.0394 (12)	0.0480 (13)	0.0006 (9)	0.0031 (10)	−0.0017 (10)
C16	0.0460 (13)	0.0580 (16)	0.0401 (12)	0.0037 (11)	−0.0090 (10)	−0.0058 (11)
C17	0.0580 (15)	0.0550 (15)	0.0334 (11)	0.0133 (12)	−0.0073 (10)	0.0040 (10)
C18	0.0435 (11)	0.0386 (11)	0.0342 (10)	0.0101 (9)	0.0018 (9)	0.0101 (8)
C19	0.0295 (9)	0.0292 (9)	0.0291 (9)	0.0067 (7)	0.0025 (7)	0.0043 (7)
C20	0.0292 (8)	0.0275 (8)	0.0314 (9)	0.0061 (7)	0.0013 (7)	0.0062 (7)
C21	0.0280 (8)	0.0303 (9)	0.0302 (9)	0.0071 (7)	0.0011 (7)	0.0075 (7)
C22	0.0347 (10)	0.0341 (9)	0.0322 (9)	0.0096 (8)	0.0021 (7)	0.0110 (8)
C23	0.0360 (10)	0.0404 (10)	0.0294 (9)	0.0111 (8)	0.0018 (8)	0.0091 (8)
C24	0.0308 (9)	0.0338 (9)	0.0287 (9)	0.0061 (7)	0.0016 (7)	0.0103 (7)
C25	0.0339 (10)	0.0354 (10)	0.0359 (10)	0.0063 (8)	0.0072 (8)	0.0132 (8)
C26	0.081 (2)	0.0467 (14)	0.0545 (16)	0.0060 (14)	0.0123 (14)	0.0285 (12)

### Geometric parameters (Å, º)

**Table:**

S1—O1	1.4323 (18)	S3—O8	1.431 (2)
S1—O2	1.4361 (18)	S3—O7	1.4338 (19)
S1—N1	1.6507 (19)	S3—N4	1.649 (2)
S1—C1	1.763 (2)	S3—C14	1.763 (2)
S2—C10	1.697 (2)	S4—C23	1.700 (2)
S2—C11	1.716 (2)	S4—C24	1.719 (2)
N1—C7	1.308 (3)	N4—C20	1.310 (3)
N2—C7	1.347 (3)	N5—C20	1.343 (3)
N2—C8	1.399 (3)	N5—C21	1.400 (2)
N2—H2A	0.88 (3)	N5—H5A	0.84 (3)
N3—O3	1.217 (3)	N6—O9	1.222 (3)
N3—O4	1.224 (3)	N6—O10	1.228 (3)
N3—C10	1.443 (3)	N6—C23	1.442 (3)
O5—C12	1.221 (3)	O11—C25	1.205 (3)
O6—C12	1.322 (3)	O12—C25	1.334 (3)
O6—C13	1.450 (3)	O12—C26	1.449 (3)
C1—C2	1.381 (3)	C14—C19	1.381 (3)
C1—C6	1.392 (3)	C14—C15	1.388 (3)
C2—C3	1.391 (4)	C15—C16	1.383 (4)
C2—H2	0.9300	C15—H15	0.9300
C3—C4	1.392 (5)	C16—C17	1.386 (4)
C3—H3	0.9300	C16—H16	0.9300
C4—C5	1.389 (4)	C17—C18	1.395 (3)
C4—H4	0.9300	C17—H17	0.9300
C5—C6	1.389 (3)	C18—C19	1.386 (3)
C5—H5	0.9300	C18—H18	0.9300
C6—C7	1.484 (3)	C19—C20	1.490 (3)
C8—C11	1.394 (3)	C21—C24	1.383 (3)
C8—C9	1.412 (3)	C21—C22	1.423 (3)
C9—C10	1.365 (3)	C22—C23	1.364 (3)
C9—H9	0.9300	C22—H22	0.9300
C11—C12	1.467 (3)	C24—C25	1.475 (3)
C13—H13A	0.9600	C26—H26A	0.9600
C13—H13B	0.9600	C26—H26B	0.9600
C13—H13C	0.9600	C26—H26C	0.9600
			
O1···C25^i^	2.893 (3)	C12···O9^ii^	3.063 (3)
			
O1—S1—O2	116.41 (12)	O8—S3—O7	117.16 (13)
O1—S1—N1	109.99 (11)	O8—S3—N4	109.67 (12)
O2—S1—N1	109.26 (11)	O7—S3—N4	109.93 (11)
O1—S1—C1	111.45 (11)	O8—S3—C14	110.86 (12)
O2—S1—C1	111.32 (11)	O7—S3—C14	110.92 (12)
N1—S1—C1	96.60 (9)	N4—S3—C14	96.31 (10)
C10—S2—C11	89.52 (10)	C23—S4—C24	89.12 (10)
C7—N1—S1	109.66 (14)	C20—N4—S3	109.72 (15)
C7—N2—C8	126.69 (18)	C20—N5—C21	126.90 (18)
C7—N2—H2A	119 (2)	C20—N5—H5A	118 (2)
C8—N2—H2A	115 (2)	C21—N5—H5A	114 (2)
O3—N3—O4	125.1 (2)	O9—N6—O10	124.5 (2)
O3—N3—C10	118.6 (2)	O9—N6—C23	118.5 (2)
O4—N3—C10	116.3 (2)	O10—N6—C23	117.1 (2)
C12—O6—C13	117.00 (19)	C25—O12—C26	116.8 (2)
C2—C1—C6	123.0 (2)	C19—C14—C15	122.7 (2)
C2—C1—S1	129.99 (19)	C19—C14—S3	107.78 (15)
C6—C1—S1	107.04 (14)	C15—C14—S3	129.52 (19)
C1—C2—C3	116.5 (2)	C16—C15—C14	117.0 (2)
C1—C2—H2	121.8	C16—C15—H15	121.5
C3—C2—H2	121.8	C14—C15—H15	121.5
C2—C3—C4	121.7 (2)	C15—C16—C17	120.9 (2)
C2—C3—H3	119.2	C15—C16—H16	119.5
C4—C3—H3	119.2	C17—C16—H16	119.5
C5—C4—C3	120.8 (2)	C16—C17—C18	121.7 (2)
C5—C4—H4	119.6	C16—C17—H17	119.2
C3—C4—H4	119.6	C18—C17—H17	119.2
C4—C5—C6	118.3 (2)	C19—C18—C17	117.4 (2)
C4—C5—H5	120.9	C19—C18—H18	121.3
C6—C5—H5	120.9	C17—C18—H18	121.3
C5—C6—C1	119.77 (19)	C14—C19—C18	120.26 (19)
C5—C6—C7	130.5 (2)	C14—C19—C20	109.28 (18)
C1—C6—C7	109.67 (17)	C18—C19—C20	130.45 (19)
N1—C7—N2	123.96 (18)	N4—C20—N5	123.65 (18)
N1—C7—C6	116.99 (18)	N4—C20—C19	116.87 (18)
N2—C7—C6	119.05 (18)	N5—C20—C19	119.48 (18)
C11—C8—N2	121.07 (19)	C24—C21—N5	121.09 (18)
C11—C8—C9	112.35 (18)	C24—C21—C22	112.71 (18)
N2—C8—C9	126.54 (19)	N5—C21—C22	126.20 (18)
C10—C9—C8	109.73 (19)	C23—C22—C21	108.87 (19)
C10—C9—H9	125.1	C23—C22—H22	125.6
C8—C9—H9	125.1	C21—C22—H22	125.6
C9—C10—N3	124.6 (2)	C22—C23—N6	124.6 (2)
C9—C10—S2	115.90 (17)	C22—C23—S4	116.54 (16)
N3—C10—S2	119.44 (16)	N6—C23—S4	118.88 (17)
C8—C11—C12	125.68 (19)	C21—C24—C25	126.73 (18)
C8—C11—S2	112.46 (16)	C21—C24—S4	112.76 (15)
C12—C11—S2	121.70 (16)	C25—C24—S4	120.43 (15)
O5—C12—O6	125.7 (2)	O11—C25—O12	125.3 (2)
O5—C12—C11	122.7 (2)	O11—C25—C24	123.3 (2)
O6—C12—C11	111.63 (19)	O12—C25—C24	111.37 (18)
O6—C13—H13A	109.5	O12—C26—H26A	109.5
O6—C13—H13B	109.5	O12—C26—H26B	109.5
H13A—C13—H13B	109.5	H26A—C26—H26B	109.5
O6—C13—H13C	109.5	O12—C26—H26C	109.5
H13A—C13—H13C	109.5	H26A—C26—H26C	109.5
H13B—C13—H13C	109.5	H26B—C26—H26C	109.5
			
O1—S1—N1—C7	−117.46 (18)	O8—S3—N4—C20	−113.04 (18)
O2—S1—N1—C7	113.60 (17)	O7—S3—N4—C20	116.77 (18)
C1—S1—N1—C7	−1.75 (18)	C14—S3—N4—C20	1.79 (18)
O1—S1—C1—C2	−61.9 (2)	O8—S3—C14—C19	112.61 (17)
O2—S1—C1—C2	69.9 (2)	O7—S3—C14—C19	−115.43 (16)
N1—S1—C1—C2	−176.5 (2)	N4—S3—C14—C19	−1.26 (17)
O1—S1—C1—C6	116.68 (16)	O8—S3—C14—C15	−67.5 (3)
O2—S1—C1—C6	−111.53 (16)	O7—S3—C14—C15	64.5 (3)
N1—S1—C1—C6	2.15 (16)	N4—S3—C14—C15	178.6 (2)
C6—C1—C2—C3	1.1 (4)	C19—C14—C15—C16	0.6 (4)
S1—C1—C2—C3	179.5 (2)	S3—C14—C15—C16	−179.3 (2)
C1—C2—C3—C4	1.1 (4)	C14—C15—C16—C17	−0.6 (4)
C2—C3—C4—C5	−2.2 (5)	C15—C16—C17—C18	0.2 (4)
C3—C4—C5—C6	1.0 (4)	C16—C17—C18—C19	0.3 (4)
C4—C5—C6—C1	1.1 (3)	C15—C14—C19—C18	−0.1 (3)
C4—C5—C6—C7	−177.8 (2)	S3—C14—C19—C18	179.79 (17)
C2—C1—C6—C5	−2.3 (3)	C15—C14—C19—C20	−179.5 (2)
S1—C1—C6—C5	179.00 (17)	S3—C14—C19—C20	0.4 (2)
C2—C1—C6—C7	176.9 (2)	C17—C18—C19—C14	−0.3 (3)
S1—C1—C6—C7	−1.9 (2)	C17—C18—C19—C20	178.9 (2)
S1—N1—C7—N2	−178.88 (17)	S3—N4—C20—N5	177.40 (17)
S1—N1—C7—C6	0.9 (2)	S3—N4—C20—C19	−1.9 (2)
C8—N2—C7—N1	−1.7 (4)	C21—N5—C20—N4	1.5 (3)
C8—N2—C7—C6	178.53 (19)	C21—N5—C20—C19	−179.26 (18)
C5—C6—C7—N1	179.8 (2)	C14—C19—C20—N4	0.9 (3)
C1—C6—C7—N1	0.8 (3)	C18—C19—C20—N4	−178.4 (2)
C5—C6—C7—N2	−0.5 (3)	C14—C19—C20—N5	−178.37 (19)
C1—C6—C7—N2	−179.47 (19)	C18—C19—C20—N5	2.3 (3)
C7—N2—C8—C11	167.2 (2)	C20—N5—C21—C24	172.4 (2)
C7—N2—C8—C9	−15.3 (4)	C20—N5—C21—C22	−7.9 (3)
C11—C8—C9—C10	0.1 (3)	C24—C21—C22—C23	−0.2 (3)
N2—C8—C9—C10	−177.6 (2)	N5—C21—C22—C23	−179.8 (2)
C8—C9—C10—N3	177.6 (2)	C21—C22—C23—N6	178.6 (2)
C8—C9—C10—S2	−1.4 (3)	C21—C22—C23—S4	0.0 (2)
O3—N3—C10—C9	−9.3 (4)	O9—N6—C23—C22	4.9 (4)
O4—N3—C10—C9	171.6 (2)	O10—N6—C23—C22	−174.4 (2)
O3—N3—C10—S2	169.59 (19)	O9—N6—C23—S4	−176.59 (19)
O4—N3—C10—S2	−9.5 (3)	O10—N6—C23—S4	4.1 (3)
C11—S2—C10—C9	1.7 (2)	C24—S4—C23—C22	0.10 (18)
C11—S2—C10—N3	−177.3 (2)	C24—S4—C23—N6	−178.52 (18)
N2—C8—C11—C12	3.4 (3)	N5—C21—C24—C25	3.2 (3)
C9—C8—C11—C12	−174.5 (2)	C22—C21—C24—C25	−176.52 (19)
N2—C8—C11—S2	178.97 (16)	N5—C21—C24—S4	179.92 (15)
C9—C8—C11—S2	1.1 (2)	C22—C21—C24—S4	0.2 (2)
C10—S2—C11—C8	−1.53 (18)	C23—S4—C24—C21	−0.20 (17)
C10—S2—C11—C12	174.2 (2)	C23—S4—C24—C25	176.80 (18)
C13—O6—C12—O5	0.1 (4)	C26—O12—C25—O11	6.7 (4)
C13—O6—C12—C11	−179.2 (2)	C26—O12—C25—C24	−173.1 (2)
C8—C11—C12—O5	0.2 (4)	C21—C24—C25—O11	9.4 (4)
S2—C11—C12—O5	−175.0 (2)	S4—C24—C25—O11	−167.15 (19)
C8—C11—C12—O6	179.5 (2)	C21—C24—C25—O12	−170.9 (2)
S2—C11—C12—O6	4.3 (3)	S4—C24—C25—O12	12.6 (3)

Symmetry codes: (i) −*x*+1, −*y*, −*z*+1; (ii) −*x*+2, −*y*, −*z*+1.

### Hydrogen-bond geometry (Å, º)

**Table:**

*D*—H···*A*	*D*—H	H···*A*	*D*···*A*	*D*—H···*A*
N2—H2*A*···O5	0.88 (3)	2.08 (3)	2.765 (2)	134 (3)
N5—H5*A*···O11	0.84 (3)	2.17 (3)	2.825 (3)	134 (3)
N5—H5*A*···O2^ii^	0.84 (3)	2.55 (3)	3.141 (3)	128 (3)
C4—H4···O4^iii^	0.93	2.48	3.376 (3)	162
C5—H5···O10^iii^	0.93	2.51	3.316 (3)	146
C17—H17···O3^iv^	0.93	2.38	3.223 (3)	151

Symmetry codes: (ii) −*x*+2, −*y*, −*z*+1; (iii) *x*+1, *y*+1, *z*; (iv) *x*+1, *y*, *z*+1.
